# 683. Epidemiology and Microbiology of Infective Endocarditis: A Six Year Experience

**DOI:** 10.1093/ofid/ofab466.880

**Published:** 2021-12-04

**Authors:** Niyas Vettakkara Kandy Muhammed, Rajalakshmi Ananthanarayanan, Aswathy Sasidharan

**Affiliations:** Kerala Institute of Medical Sciences, Trivandrum, Kerala, India

## Abstract

**Background:**

The epidemiology and microbiology of infective endocarditis (IE) is not well studied in India. Studies from developed countries report a culture positivity of more than 90% in IE, while in India it has been lower (40–70%). Viridans Group Streptococci (VGS) are the commonest organism identified from previous Indian studies. The state of Kerala in India has better health indicators compared to the rest of India and it is likely that the epidemiology of IE in Kerala may be different. We therefore studied the epidemiology and microbiology of IE in patients admitted to a tertiary care hospital in Kerala over six years (2015 – 2020).

**Methods:**

An electronic medical record search was conducted to identify patients who satisfied definite or possible IE criteria as per modified Duke criteria. Three sets of blood cultures were sent in BacT/Alert blood culture bottles for all suspected cases of IE. Blood culture was done using BacT-ALERT 3D automated microbial detection system (bioMérieux, France) and organisms were identified using VITEK-2 system. Transthoracic echocardiogram was done for all patients and a transoesophageal echocardiogram was done when indicated.

**Results:**

70 patients satisfied the inclusion criteria. Majority (70.4%) were male; mean age was 50.7±16.3 years. 71% patients had underlying valvular heart disease. Diabetes mellitus (53.5%) was the most common comorbidity followed by chronic kidney disease (18.3%). Mitral valve was most commonly affected (53.5%) followed by the aortic valve (19.7%) and both valves were involved in 5.7%. Right sided valves were affected in 8.5%. Prosthetic valve endocarditis accounted for 10% of cases. No echocardiographic evidence of endocarditis was seen in 11.3%. Blood culture was positivity was 64.8%. Staphylococcus aureus (20%) was the most common organism isolated, followed by VGS (17.1%). 50% of the Staphylococcus aureus isolated were methicillin resistant. Among 57 patients in whom an outcome was recorded, mortality was 12.2%.

Microbiology profile of infective endocarditis

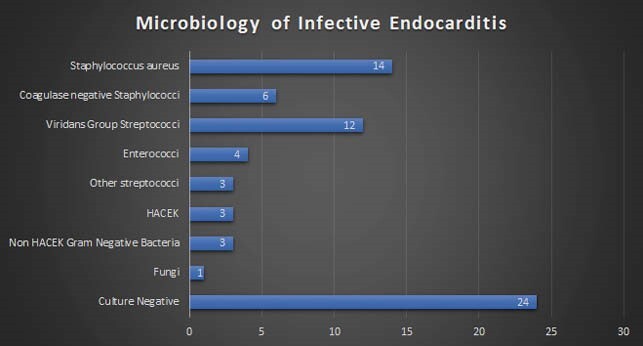

**Conclusion:**

Staphylococcus aureus has emerged as the most common etiological agent of IE in our study, in contrast to previous studies from India where VGS was predominant. The high prevalence of MRSA is of concern.

**Disclosures:**

**All Authors**: No reported disclosures

